# Pyroptosis: A Promising Mechanism Linking SARS-CoV-2 Infection to Adverse Pregnancy Outcomes

**DOI:** 10.3390/ijms24119278

**Published:** 2023-05-25

**Authors:** Paola Monti, Giulia Solazzo, Veronica Accurti, Bianca Gambitta, Simona Iodice, Simona Boito, Laura Cantone, Alessandro Manenti, Laura Dioni, Emanuele Montomoli, Nicola Persico, Valentina Bollati

**Affiliations:** 1 EPIGET Lab, Department of Clinical Sciences and Community Health, University of Milan, 20122 Milan, Italy; paola.monti@unimi.it (P.M.); giulia.solazzo@unimi.it (G.S.); simona.iodice@unimi.it (S.I.); laura.cantone@unimi.it (L.C.); laura.dioni@unimi.it (L.D.); 2 Fetal Medicine and Surgery Service, Fondazione IRCCS Ca’ Granda, Ospedale Maggiore Policlinico, 20122 Milan, Italy; veronica.accurti@gmail.com (V.A.); b.gambitta@gmail.com (B.G.); simona.boito@gmail.com (S.B.); 3 VisMederi Srl, 53100 Siena, Italy; alessandro.manenti@vismederi.com (A.M.); emanuele.montomoli@unisi.it (E.M.); 4 Department of Molecular and Developmental Medicine, University of Siena, 53100 Siena, Italy; 5 Department of Clinical Sciences and Community Health, University of Milan, 20122 Milan, Italy; 6 CRC, Center for Environmental Health, University of Milan, 20122 Milan, Italy; 7 Occupational Health Unit, Fondazione IRCCS Ca’ Granda-Ospedale Maggiore Policlinico, 20122 Milan, Italy

**Keywords:** SARS-CoV-2, COVID-19, neutralizing antibodies, pregnancy, miRNA, NLRP3, pyroptosis, pre-eclampsia, gestational diabetes, abnormal fetal growth

## Abstract

Pregnancy is characterized by a delicate immune balance; therefore, infectious diseases might increase the risk of adverse pregnancy outcomes (APOs). Here, we hypothesize that pyroptosis, a unique cell death pathway mediated by the NLRP3 inflammasome, could link SARS-CoV-2 infection, inflammation, and APOs. Two blood samples were collected from 231 pregnant women at 11–13 weeks of gestation and in the perinatal period. At each time point, SARS-CoV-2 antibodies and neutralizing antibody titers were measured by ELISA and microneutralization (MN) assays, respectively. Plasmatic NLRP3 was determined by ELISA. Fourteen miRNAs selected for their role in inflammation and/or pregnancy were quantified by qPCR and further investigated by miRNA-gene target analysis. NLRP3 levels were positively associated with nine circulating miRNAs, of which miR-195-5p was increased only in MN+ women (*p*-value = 0.017). Pre-eclampsia was associated with a decrease in miR-106a-5p (*p*-value = 0.050). miR-106a-5p (*p*-value = 0.026) and miR-210-3p (*p*-value = 0.035) were increased in women with gestational diabetes. Women giving birth to small for gestational age babies had lower miR-106a-5p and miR-21-5p (*p*-values = 0.001 and 0.036, respectively), and higher miR-155-5p levels (*p*-value = 0.008). We also observed that neutralizing antibodies and NLRP3 concentrations could affect the association between APOs and miRNAs. Our findings suggest for the first time a possible link between COVID-19, NLRP3-mediated pyroptosis, inflammation, and APOs. Circulating miRNAs might be suitable candidates to gain a comprehensive view of this complex interplay.

## 1. Introduction

Pregnancy is a unique immunological condition characterized by the alternation of pro- and anti-inflammatory states throughout different gestational stages [1]. In addition, immune responses must be finely tuned to guarantee protection against infections and endogenous stressors while at the same time maintaining tolerance toward the fetus [2,3]. Such a delicate equilibrium is achieved through a series of complex adaptations in the maternal immune system [3], whose perturbation could result in an increased risk of adverse pregnancy outcomes (APOs) in terms of maternal, fetal, and neonatal complications. In particular, APOs such as pre-eclampsia, gestational diabetes mellitus, and abnormal fetal growth have been reported to occur more frequently in women with autoimmune disorders [4,5,6] or experiencing viral or bacterial infections during pregnancy [7,8,9].

In this scenario, the SARS-CoV-2 pandemic outbreak has raised concerns for expectant mothers. Indeed, infectious diseases caused by other coronaviruses have been previously associated with increased vulnerability to APOs [10]. Although the possible association between COVID-19 and APOs remains controversial [10,11,12], these two conditions have been hypothesized to share some biological processes such as endothelial dysfunction and immune deregulation [13]; nevertheless, knowledge on this topic is still limited. One of the mechanisms potentially linking SARS-CoV-2 infection, immune response, and APOs is pyroptosis, a unique cell death mode that can be triggered by many pro-inflammatory cues, resulting in the activation of pattern recognition receptors and the inflammasome assembly [14]. In particular, the NLRP3 inflammasome/caspase-1 pathway promotes the cleavage of gasdermins, a family of proteins that oligomerize and form pores on the plasma membrane, causing cell swelling and osmotic lysis. Pyroptosis also results in the extracellular leakage of many pro-inflammatory cytokines (e.g., IL-1β and IL-18), which foster the spreading of inflammation [14]. Of note, the NLRP3 inflammasome itself can be released in the extracellular space during the pyroptotic process [15].

Although pyroptosis is thought to be an intrinsically protective mechanism, its exacerbation can lead to excessive inflammation and tissue damage [16]. Such a “double-edged” role has been reported both in patients with COVID-19 [17,18] and in pregnancy [19,20]. Nevertheless, the role of pyroptosis in pregnant women infected by SARS-CoV-2 remains largely uninvestigated. Interestingly, many studies have reported that the NLRP3 signaling cascade can be controlled by miRNAs, either originating in the cell itself or internalized from the extracellular space [21,22,23]. In addition, miRNAs might be released as a consequence of pyroptosis, posing as damage-associated molecular patterns (DAMPs) [24]. In this scenario, the molecular phenotyping of circulating miRNAs could help shed light on COVID-19 pathophysiology in pregnant women, especially for the identification of biological factors underlying the increased risk of APOs.

In this study, we analyzed a population of 231 pregnant women who were followed up from the first trimester of gestation until delivery. Blood samples collected between 11 and 13 weeks of gestation (T0) and in the perinatal period (T1) were used to measure SARS-CoV2 immunoglobulins and neutralizing antibodies. We also quantified cell-free NLRP3 inflammasome and 14 miRNAs selected for their role in inflammation and pregnancy, as reported in literature. All data collected were elaborated by statistical analysis to investigate the possible correlation between SARS-CoV-2 infection, inflammation, and APOs.

## 2. Results

### 2.1. Characteristics of the Study Population

Study participants (Supplementary Table S1) are a subgroup of a larger population recruited in previous research (*n* = 528), from which 104 women were selected for their positivity to SARS-CoV2 antibodies and 127 as matched negative controls. The majority of positive women were asymptomatic or had mild symptoms. Among the 231 pregnant women enrolled in this study, the average maternal age was 33.6 years (SD = 4.7 years), with no significant difference between SARS-CoV2 Ig-negative and positive ones. Instead, a slight difference was observed regarding maternal weight (*p*-value = 0.049), with Ig-positive women having higher Body Mass Index (BMI) values than negative ones. Only a minority of women were smokers (4.3%), while 89.6% never smoked and 6.1% quit smoking at the beginning of pregnancy. About two-thirds of study participants (64.1%) were in their first pregnancy.

Most women (86.1%) had a pregnancy without complications, with both the mean gestational age at delivery (39.3 ± 1.3 weeks) and the mean neonatal weight (3312.0 ± 432.9 g) being in normal ranges. However, 32 women (13.9%) experienced one or more adverse pregnancy outcomes, the most frequent being abnormal fetal growth (17 cases), with 6 small for gestational age (SGA) and 11 large for gestational age (LGA) newborns. Besides, 11 cases of gestational diabetes, 5 cases of pre-eclampsia, and 1 case of premature delivery (<34 weeks) were reported. Four newborns were also hospitalized in Neonatal Intensive Care Unit (NICU).

### 2.2. Immunoglobulin Assessment and NLRP3 Quantification

Blood samples collected at T0 and T1 were used to determine IgG, IgM, and IgA plasma concentrations by ELISA. For all the participants with a positive IgG result, a plasma aliquot was used to carry out a microneutralization (MN) assay.

As shown in Table 1, during the first trimester of pregnancy, 40 women were IgG-positive, with 27 of them (67.5%) having neutralizing anti-SARS-CoV-2 antibodies. For women showing a positive MN assay, the neutralization titer was calculated by testing six different plasma dilutions (1/10-20-40-80-160-320). All of them had an MN assay titer ranging from 1/10 to 1/80. Among the 40 IgG-positive women, 12 (30.0%) also tested positive for IgM and 3 (7.5%) for IgA.

An Ig measure carried out on peripartum samples revealed that the number of participants that were Ig-positive rose to 97, while 127 remained negative (55.0%). Among the former, 64 women (27.7%) were infected by SARS-CoV-2 between T0 and T1 (“positivized”). Instead, seven women (3.0%) that were Ig-positive at T0 became negative at T1 (“negativized”).

Considering women with anti-SARS-CoV-2 antibodies at T1, 79 (81.4%) tested positive for the MN assay, with the great majority (95.0%) having an MN assay titer between 1/10 and 1/80. 52 out of the 97 IgG-positive women (53.6%) were also positive for IgM, while IgA positivity was detected in 23 (10.0%) of them.

Plasma samples were also used to measure the concentration of the NLRP3 inflammasome. There was no significant difference between the mean concentration value measured at T0 and at T1 (*p*-value = 0.288), for women with and without APOs (*p*-value = 0.615), for women with and without any positivity for IgG, IgA, or IgM (*p*-value = 0.938), and for women with and without positive MN assays (*p*-value = 0.814) (Supplementary Table S2). Instead, we observed a significant difference regarding plasmatic miRNA levels between cases and controls. Women with any Ig positivity at T0 and/or T1 had lower mean miRNA levels of miR-155-5p (log_2_(RQ) = 0.9 vs. 1.1; *p* = 0.047) and miR-221-3p (log_2_(RQ) = 3.07 vs. 3.82, *p* = 0.037) if compared to controls (Supplementary Table S3).

### 2.3. Association of NLRP3, Ig, and MN with miRNA Expression

In order to determine if plasma NLRP3, immunoglobulins, and MN titers were associated with the expression levels of the 14 miRNAs assayed, we applied a multivariable mixed model for repeated measures adjusted for time, gestational age at sampling, and maternal age. miR-137 was excluded from statistical analysis as it was expressed at detectable levels in a very low proportion of plasma samples (2.6%).

**Figure 1:**
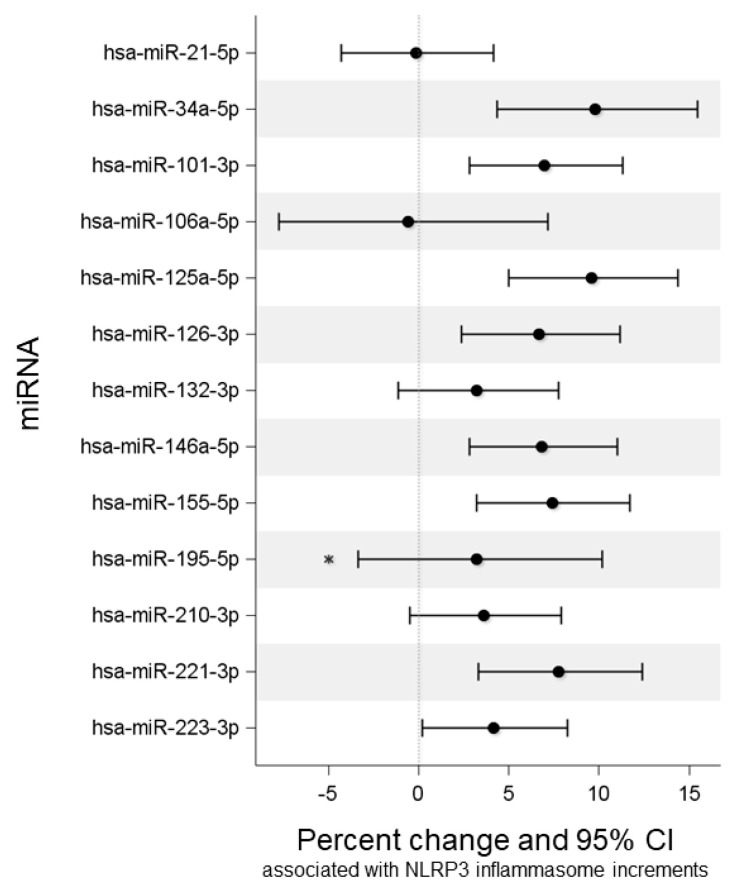
Percentage changes in miRNA expression levels associated with NLRP3 inflammasome increments. Multivariable linear mixed model for repeated measures adjusted for time, gestational age at sampling, and maternal age. miRNAs are expressed as log_2_(RQ). Percentage change is calculated as (2^(β)^ − 1) × 100 and corresponds to the percentage change in miRNA expression level associated with one unit increase in the NLRP3 inflammasome. The asterisk indicates a significant *p*-value (*p* = 0.018) of the interaction between NLRP3 and MN when we added the interaction to the previous model. CI, confidence interval.

The percentage changes in miRNA expression levels associated with NLRP3 unitary increments are shown in Figure 1.

As reported in Table 2, plasma NLRP3 inflammasome increments were associated with increased expression of eight miRNAs, i.e., miR-34a-5p (*p*-value < 0.001), miR-101-3p (*p*-value = 0.001), miR-125a-5p (*p*-value < 0.001), miR-126-3p (*p*-value = 0.002), miR-146a-5p (*p*-value = 0.001), miR-155-5p (*p*-value < 0.001), miR-221-3p (*p*-value = 0.001), and miR-223-5p (*p*-value = 0.039). Interestingly, none of the selected miRNAs were found to be downregulated in response to NLRP3 increases.

**Figure 2:**
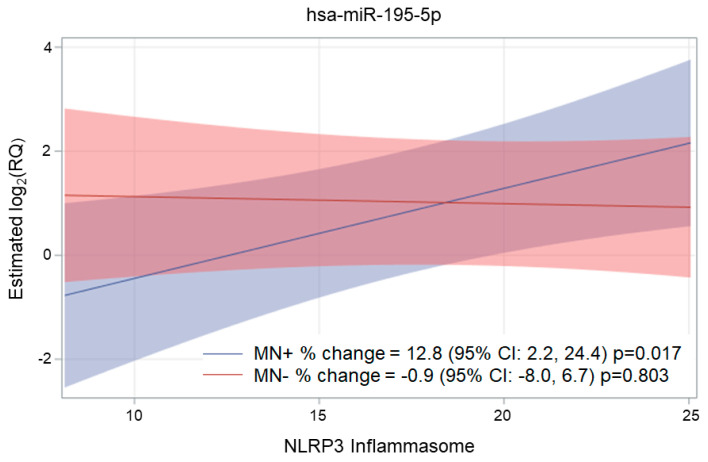
Interaction of the NLRP3 inflammasome with microneutralization at the miR-195-5p expression level. Estimates from a multivariable linear mixed model for repeated measures are adjusted for time, gestational age at the sample, MN, the interaction between NLRP3 inflammasome and MN, and maternal age. miR-195-5p is expressed as log_2_(RQ). Estimates for plot purposes were calculated at the mean age of 33.6 and the mean gestational age of 24.2 during peripartum.

Although NLRP3 increments alone were not associated with changes in miR-195-5p expression levels, we observed that the interaction product between NLRP3 and MN was associated with miR-195-5p (*p*-value of the interaction = 0.018). In particular, increasing plasma NLRP3 concentrations were found to be positively associated with miR-195-5p levels (*p*-value = 0.017) in women with neutralizing antibodies (MN+), whereas no trend (*p*-value = 0.803) was observed for MN-study participants (Figure 2).

No significant association was found between IgG, IgM, and IgA and selected miRNAs (see Supplementary Tables S4–S6), nor between MN titer and miRNAs (Supplementary Table S7).

### 2.4. Effect of Adverse Pregnancy Outcomes on miRNA Expression

**Figure 3:**
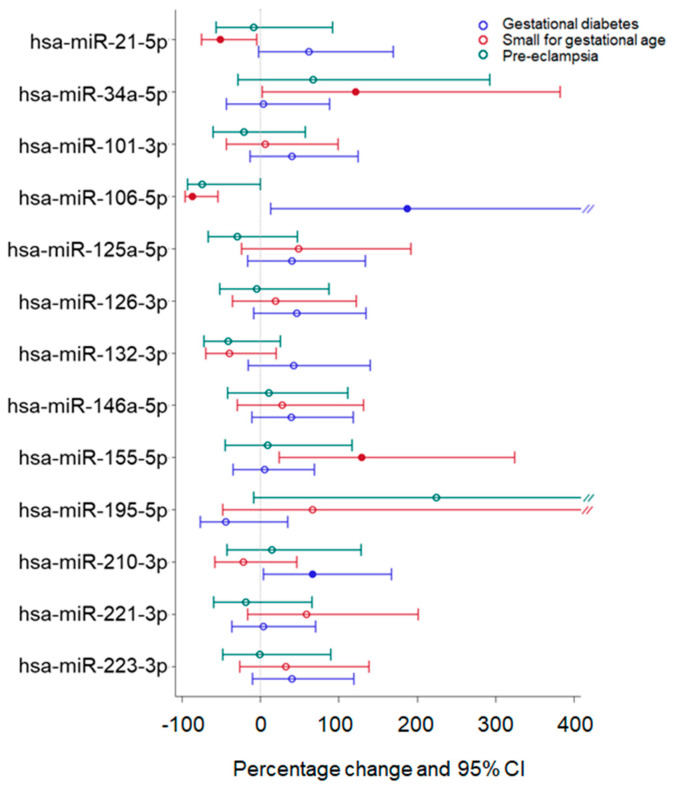
Percentage change of miRNA expression level in women having an adverse outcome during pregnancy. The X-axis represents the percentage change of miRNA in women having an adverse outcome during pregnancy (gestational diabetes, small for gestational age, pre-eclampsia) versus women not experiencing it. Multivariable linear mixed models for repeated measures were adjusted for time, gestational age at the sample, and maternal age. miRNAs are expressed as log_2_(RQ). Percentage change is calculated as (2^(β)^ − 1) × 100. Solid-colored dots represent significant percentage changes.

We then evaluated whether women who experienced adverse pregnancy outcomes (APOs) had different plasmatic miRNA levels compared to women without pregnancy complications. APOs were considered either as single maternal/neonatal complications or as combined outcomes (composite adverse outcomes). As shown in Figure 3, pre-eclampsia was associated with a borderline decrease in miR-106a-5p (*p*-value = 0.050). On the contrary, the same miRNA (*p*-value = 0.026) and miR-210-3p (*p*-value = 0.035) were increased in women with gestational diabetes. In addition, three miRNAs were found to be differentially expressed in women giving birth to SGA babies: while miR-106a-5p and miR-21-5p were found to be decreased (*p*-values = 0.001 and 0.036, respectively), this neonatal condition was associated with higher miR-155-5p levels (*p*-value = 0.008).

Instead, no association was found between composite adverse outcomes, abnormal growth, or LGA and miRNA expression levels (Supplementary Table S8).

**Figure 4:**
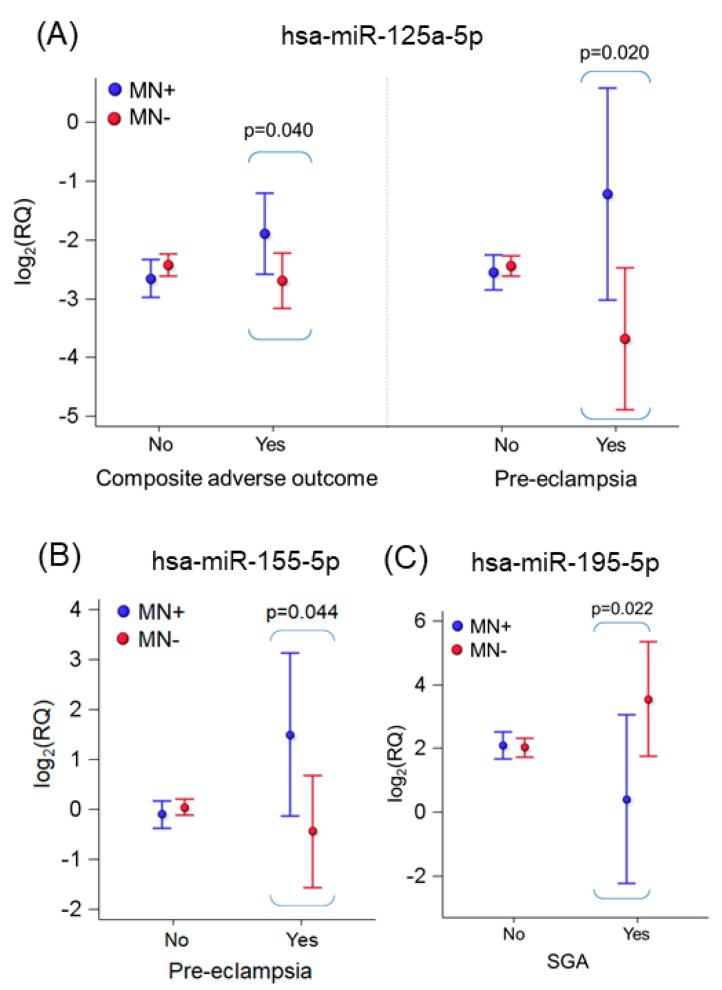
Interaction of MN with selected APOs in the expression levels of miR-125a-5p (**A**), miR-155-5p (**B**), and miR-195-5p (**C**). Estimates from a multivariable mixed model for repeated measures adjusted for time, gestational age at the sample, MN, adverse outcome, interaction between APOs and MN, and maternal age miRNAs are expressed as log_2_(RQ). The *p*-value refers to the difference between MN+ and MN− women experiencing the APOs. Estimates for plot purposes were calculated at the mean age of 33.6 and the mean gestational age of 24.2 during peripartum.

When testing the possible effect of the interaction between MN and APOs on miRNA levels, we observed changes in three miRNAs, i.e., miR-125a-5p, miR-155-5p, and miR-195-5p (Figure 4). In detail, miR-125a-5p levels were higher in MN+ women that experienced composite adverse outcomes (*p*-value of the interaction = 0.015). Differences for single adverse outcomes are also reported for miR-125a-5p and miR-155-5p (pre-eclampsia×MN) and for miR-195-5p (SGA×MN). For complete data, see Supplementary Table S9.

**Figure 5:**
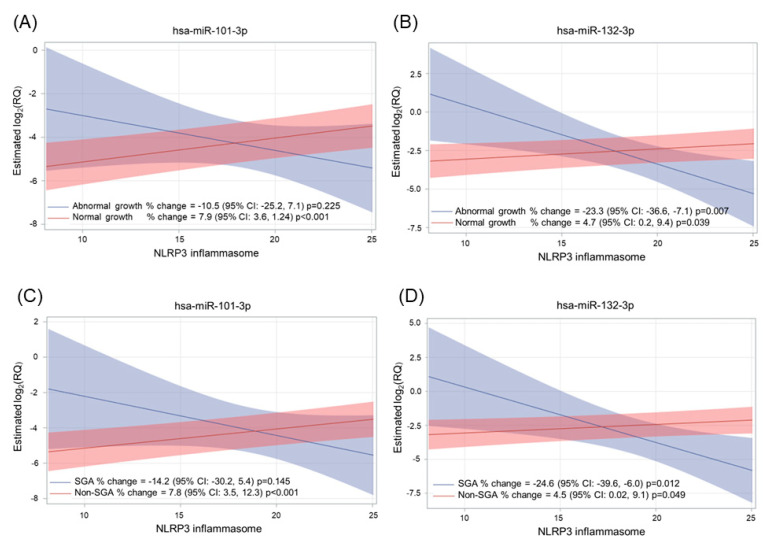
Interaction of the NLRP3 inflammasome with APOs on the expression levels of miR-101-3p and miR-132-3p. Upper panels show the interaction between NLRP3 and abnormal growth in affecting miR-101-3p (**A**) and miR-132-3p (**B**); lower panels show the interaction between NLRP3 and SGA in affecting miR-101-3p (**C**) and miR-132-3p (**D**). Estimates from a multivariable linear mixed model for repeated measures are adjusted for time, gestational age at the sample, NLRP3 inflammasome, maternal age, and the interaction between NLRP3 inflammasome and adverse outcome. miRNAs are expressed as log2(RQ). Percentage change is calculated as (2^(β)^ − 1) × 100. Estimates for plot purposes were calculated at the mean age of 33.6 years and the mean gestational age of 24.2 weeks during peripartum.

Finally, we evaluated the effect of the interaction between plasma NLRP3 concentrations and APOs on miRNA levels. We observed that two miRNAs, i.e., miR-101-3p and miR-132-3p, were differentially expressed in women with single APOs in response to NLRP3 increments (Figure 5). Specifically, NLRP3 concentration was associated with miR-101-3p expression only in women giving birth to newborns with normal growth (*p*-value < 0.001) and in non-SGA pregnancies (*p*-value < 0.001). Instead, NLRP3 increments were associated with decreased miR-132-3p expression in abnormal growth (*p*-value = 0.007) and SGA cases (*p*-value = 0.012). miR-132-3p followed the opposite trend in normal growth (*p*-value = 0.039) and non-SGA pregnancies (*p*-value = 0.049). For complete data, see Supplementary Table S10.

### 2.5. Bioinformatic Analysis

In order to investigate the potential impact of miRNA changes on gene expression, we performed a bioinformatics analysis to identify the target genes of the eight miRNAs that we found to be associated with plasma NLRP3 increments (as shown in Figure 2 and Table 2; i.e., miR-101-3p, miR-125a-5p, miR-126-3p, miR-146a-5p, miR-155-5p, miR-221-3p, miR-223-5p, and miR-34a-5p). We also included miR-195-5p since NLRP3 increments were positively associated with this miRNA in MN+ women. Target genes of these nine miRNAs are reported in Supplementary Table S11.

The target genes reported in Supplementary Table S11 were then compared with genes associated with inflammation (*n* = 467), as reported in the DisGeNET database. We found that eight out of the nine miRNAs of interest target genes are associated with inflammation. The only exception was miR-126-3p, which did not target any inflammation-related genes. In total, we found 47 genes associated with inflammation and targeted by at least one of the eight miRNAs (Supplementary Table S12).

**Figure 6:**
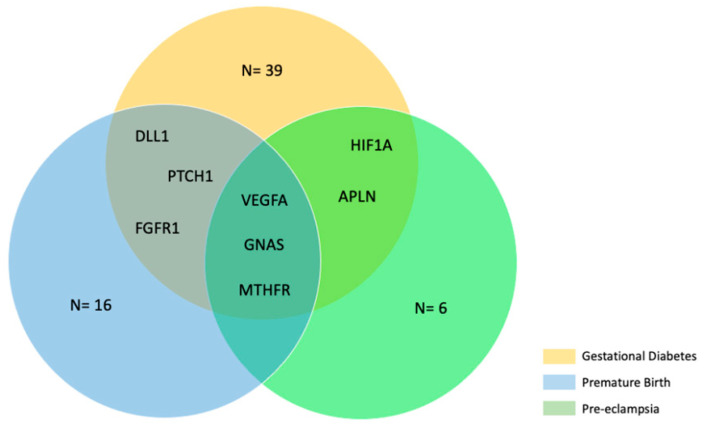
Shared target genes among gestational diabetes, premature birth, and pre-eclampsia.

Then, we used data obtained from the gene target prediction analysis (Supplementary Table S11) to further analyze the three miRNAs that we found to be associated with MN*APOs, i.e., miR-125a-5p, miR-155-5p, and miR-195-5p. We compared the genes targeted by these three miRNAs with genes related to gestational diabetes (*n* = 649), premature birth (*n* = 192), and pre-eclampsia (*n* = 166), as reported in DisGeNET (v7.0) datasets. We found that 47 gene targets were associated with gestational diabetes, 22 with premature birth, and 11 with pre-eclampsia (Supplementary Table S13). Among these genes, some were shared between the three disease datasets (Figure 6).

**Figure 7:**
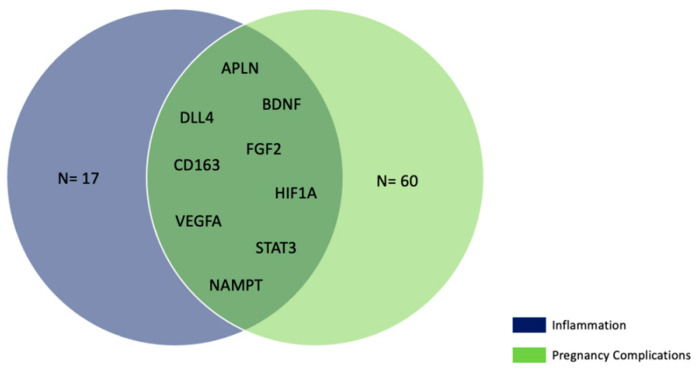
Target genes implied in either inflammation and/or pregnancy complications.

Finally, we compared the 26 inflammation genes targeted by miR-125a-5p, miR-155-5p, and miR-195-5p with the 69 genes associated with pregnancy complications (i.e., gestational diabetes, premature birth, and pre-eclampsia). We found that nine genes (Supplementary Table S14, Figure 7) targeted by the three miRNAs were involved in either inflammatory processes or APOs.

## 3. Discussion

In the present study, conducted on 231 pregnant women, we aimed to gain a comprehensive view of the complex interplay between SARS-CoV-2 infection, inflammation, and pregnancy outcomes.

Pyroptosis is a peculiar lytic cell death mode was been first described in the 1990s as a process occurring in macrophages after infection by Gram-negative *Shigella flexneri* [25]. According to our initial hypothesis that pyroptosis could be a potential mechanism mediating the effect of SARS-CoV-2 infection on pregnancy, we measured the plasmatic concentration of the NLRP3 inflammasome, a multi-protein complex mainly expressed in monocytes/macrophages [26]. Within the cell, NLRP3 inflammasome assembly is triggered by the oligomerization of NLRP3, a cytoplasmic pattern-recognition receptor, upon a variety of stress signals (both endogenous and exogenous), including viral infections; once assembled, the NLRP3 inflammasome recruits and promotes the autocatalytic activation of caspase-1, which in turn starts a signaling cascade eventually culminating in pyroptotic cell death [27]. NLRP3-mediated pyroptosis poses as a crucial component of the innate immune response, not only does it cause the ejection of viral particles/components, thus blocking their intracellular replication and promoting their recognition by immune cells, but it also fosters the recruitment and activation of immune cells through the release of many pro-inflammatory factors [18]. During this process, the NLRP3 inflammasome itself can be released outside the cell to propagate inflammation [28], probably through an active mechanism mediated by extracellular vesicles (EVs) [15].

First, we selected 14 candidate miRNAs already known to participate in inflammation and/or pregnancy, and we observed that NLRP3 increments were associated with increased expression levels of plasmatic miR-101-3p (*p*-value = 0.001), miR-125a-5p (*p*-value < 0.001), miR-126-3p (*p*-value = 0.002), miR-146a-5p (*p*-value = 0.001), miR-155-5p (*p*-value < 0.001), miR-221-3p (*p*-value = 0.001), miR-223-5p (*p*-value = 0.039), and miR-34a-5p (*p*-value < 0.001). According to bioinformatic analysis, all these miRNAs target genes involved in different stages of the inflammatory process, from sensing inflammatory cues to signal transduction and response. Notably, some of these miRNAs (i.e., miR-34a, miR-146a-5p, and miR-125a-5p) are known to suppress pyroptosis by targeting the *NLRP3* gene [29,30,31]; on the contrary, miR-155-5p exerts the opposite effect, as its inhibition has been associated with *NLRP3* downregulation [32]. Since there is growing evidence supporting that inflammasome activation correlates with increased shedding of EVs [33], it is possible that the general increase in circulating miRNAs we observed was a result of their role in EV molecular cargo. Nevertheless, miRNAs are likely to be loaded within EVs through specific sorting mechanisms [34], thus suggesting that their release could be part of a specific response to inflammasome activation.

Regarding miR-195-5p, we observed that its expression levels were positively associated with NLRP3 concentration only in women with neutralizing antibodies (MN+). The presence of neutralizing antibodies is often regarded as an indicator of an effective and robust immune response against viral infections. However, they have also been associated with COVID-19 severity [35]. miR-195-5p is already known for its role in SARS-CoV-2 infection; indeed, it has been proposed as part of a three-miRNA signature capable of discriminating with 99.9% accuracy between COVID-19 patients and healthy controls [36]. Besides, this miRNA has been predicted to directly bind to the viral RNA of human coronaviruses [37,38] and was found to be strongly upregulated in the lungs of hamsters after SARS-CoV-2 infection [38]. Although miR-195-5p has never been linked to pyroptosis, its overexpression is known to induce apoptosis, thus preventing excessive proliferation of infected cells and hampering the spreading of the infection [38]. In this context, it is possible that the increased expression levels of this plasma miRNA observed in response to NLRP3 increments might be part of an anti-viral defense mechanism occurring only in women with a robust immune response (MN+); however, extensive cell death could also lead to tissue damage, thus exacerbating disease severity. Further experimental studies are needed to explore these hypotheses.

The profile of circulating miRNAs was also found to be altered in women experiencing APOs. In this regard, we observed that pre-eclampsia was associated with decreased miR-106a-5p levels (*p*-value = 0.050). As miR-106a-5p is highly expressed in villous tissues and plays a role in regulating trophoblastic angiogenesis [39], it is likely that it might be implied in the etiology of this hypertensive disorder, as also suggested by a previous transcriptomic study [40]. Moreover, we also found the same miRNA to be increased in women with gestational diabetes, along with miR-210-3p (*p*-values = 0.035 and 0.026, respectively). Although the role of miR-106a-5p in gestational diabetes has never been investigated, there is evidence of its implication in glucose homeostasis as it targets *FOXO1*, a key regulator of insulin signaling [41]. Instead, a case-control study found an association between increased levels of miR-210-3p and gestational diabetes, but only in women with overweight/obesity [42]. We also observed a decrease in miR-106a-5p levels in SGA pregnancies (*p*-value = 0.001), as well as miR-21-5p (*p*-value = 0.036); on the contrary, miR-155-5p was increased (*p*-value = 0.008). These findings are partly in accordance with existing literature, as increased blood miR-155-5p has been recently reported in women giving birth to SGA babies [43]. Instead, other considered APOs (composite adverse outcome, abnormal growth, and LGA) were not associated with plasmatic miRNA levels.

When evaluating the effect of the interaction between APOs and neutralizing antibodies on the pattern of plasmatic miRNAs, we observed that miR-125a-5p levels were higher in MN+ women that experienced pregnancy complications (*p*-value = 0.040). Differences for single adverse outcomes were also found for miR-125a-5p and miR-155-5p (pre-eclampsia×MN) and for miR-195-5p (SGA×MN). According to our bioinformatic analysis, these three miRNAs were predicted to target nine genes (*APLN*, *BDNF*, *DLL4*, *FGF2*, *CD163*, *HIF1A*, *VEGFA*, *STAT3*, and *NAMPT*) involved in both inflammation and APOs; of note, most of these genes (*APLN* [44], *BDNF* [45], *FGF2* [46], *HIF1A* [47], *VEGFA* [48], *STAT3* [49], and *NAMPT* [50]) have been previously implied in NLRP3-mediated pyroptosis. Therefore, our findings could suggest that miR-125a-5p, miR-155-5p, and miR-195-5p might be involved in a complex immune regulatory network that might influence the trajectory of gestation; nevertheless, since the number of MN- and MN+ women experiencing APOs is very small, these findings should be validated on a larger population before drawing firm conclusions. In addition, we tested whether NLRP3 and APOs could interact to determine miRNA levels. In this regard, NLRP3 increments were associated with miR-101-3p in women giving birth to children with normal growth (*p*-value < 0.001) and without SGA pregnancies (*p*-value < 0.001). miR-101-3p has been recently found to modulate the PTEN/Akt pathway, which plays a role in placental development, and to be downregulated in women with recurrent miscarriage [51]. A similar trend was observed for miR-132-3p, whose plasmatic levels were associated with NLRP3 increments only in normal growth (*p*-value = 0.039) and in non-SGA pregnancies (*p*-value = 0.049). On the contrary, as NLRP3 concentration increased, the expression of this miRNA decreased in abnormal growth and SGA pregnancies (*p*-values = 0.007 and 0.012, respectively). Mainly known for its role in inflammation, miR-132-3p has been recently implicated in APOs such as pre-term birth [52], pre-eclampsia [53], and gestational diabetes [54]. Besides, LPS-induced upregulation of this miRNA was found to promote NLRP3 activation and pyroptosis [55].

Our study has many strengths. First, pregnancies were followed up from the first trimester to postpartum, thus allowing us to thoroughly monitor the clinical status of both study participants and their children. Second, we assessed not only the presence of anti-SARS-CoV-2 immunoglobulins but also their neutralization potency, thus gaining additional information about the robustness of the antiviral humoral response. Third, we measured the circulating NLRP3 inflammasome as a proxy for the pyroptotic process, whose role in pregnant women with anti-SARS-CoV-2 immunoglobulins has never been studied before. Nevertheless, we should also account for some limitations, principally concerning the choice of miRNAs, which were selected *a priori* for their involvement in inflammation and/or pregnancy, and the limited incidence of APOs in our population (32 cases). Given the small number of women with APOs, additional research performed on larger populations will be required to test if there are significant differences between specific APOs regarding the associations presented in this study.

Also, here we focused on SARS-CoV-2 infection as a prototypical pro-inflammatory stimulus that might alter the normal trajectory of pregnancy; nevertheless, it is possible that the observed changes in circulating miRNAs and NLRP3 levels might be generic to other infections during pregnancy. Further studies will be necessary to address this hypothesis as well as verify whether such changes might occur in non-pregnant individuals (both males and females).

To exclude the possibility that the observed changes could be related to other pro-inflammatory conditions, exclusion criteria included:

Overall, our findings suggest that circulating miRNAs might be suitable candidate markers to gain a comprehensive picture of immune alterations occurring in pregnant women who have been infected by SARS-CoV-2. However, untargeted studies are needed to identify additional plasmatic miRNAs that might play a role in maternal immune regulation as well. Besides, our study suggests for the first time that NLRP3-mediated pyroptosis could be a new piece in the complex puzzle of immunological regulation in pregnant women, whose alteration can lead to impaired antiviral defense and gestational complications. Future studies are needed to collect experimental evidence about the role of pyroptosis at the interface between COVID-19 and APOs.

## 4. Materials and Methods

### 4.1. Subject Enrollment and Blood Sample Collection

The study included 231 pregnant women who were recruited at the Fetal Medicine Unit of the Policlinico Hospital in Milan (Italy), between April and September 2020. The study is a nested case-control study embedded in a larger investigation that has been previously described [56].

“Cases” were selected as all the women who were positive for antibodies to SARS-CoV-2 (at least one of IgG, IgM, or IgA) at T0 and/or T1 (*n* = 104). 127 “Controls” (negative for SARS-CoV-2 IgG, IgM, or IgA at T0 and T1) were matched to cases for maternal age.

Briefly, after signing a written informed consent, all the participants agreed to provide information about demographics, age, ethnicity, weight, method of conception, smoking status, and parity. Each woman was interviewed about the presence of COVID-19-like symptoms at the time of enrollment, at 20–22 weeks of gestation, and during the perinatal period. Data on pregnancy outcomes and neonatal parameters were collected from the hospital medical records if delivery occurred at our hospital, or by telephone interview otherwise. The diagnosis of adverse pregnancy outcomes (i.e., pre-eclampsia, gestational diabetes mellitus, abnormal growth, and delivery < 34 weeks) was made according to guidelines and as reported in [56].

For each participant, venous blood samples (7.5 mL) were drawn in EDTA tubes at two different time points (T0 and T1). T0 blood samples were collected at the time of recruitment, i.e., during the first trimester of pregnancy (11–13 gestational weeks). T1 blood samples were collected in the perinatal period (between peripartum and puerperium), between October 2020 and May 2021. Blood samples were processed by centrifugation at 1200× *g* for 15 min within 4 h of withdrawal to obtain a cell-free plasma fraction. Plasma aliquots were stored at −80 °C until use.

### 4.2. Enzyme-Linked Immunosorbent Assay (ELISA)

Immunoglobulin (Ig)G, IgM, and IgA quantification in human plasma samples was performed using an in-house ELISA RBD assay, as described in [56]. Briefly, 96-well ELISA plate coating was performed using 1 µg/mL of purified recombinant Wuhan/Ancestral SARS-CoV-2 Spike-RBD protein (Arg319-Phe541) (Sino Biological, Beijing, China). Detection of Ig was carried out by adding appropriate dilutions of goat anti-human IgG-Fc Horse Radish Peroxidase (HRP)-conjugated antibody or IgM (μ-chain) and IgA (α-chain) (Bethyl Laboratories, Montgomery, AL, USA), followed by incubation with TMB substrate (Bethyl Laboratories, Montgomery, AL, USA). Plates were read within 20 min at 450 nm with a SpectraMax ELISA plate reader (Molecular Devices, San Jose, CA, USA). The cut-off value was defined as three times the average of OD values from negative control wells (a pool of three pre-pandemic human plasma samples). Samples with ODs above the cut-off at the lowest dilution were considered Ig-positive.

Quantification of plasmatic NLRP3 was carried out using a commercial ELISA kit (cat. MBS3802246, MyBioSource, San Diego, CA, USA) according to the manufacturer’s instructions. Briefly, plasma samples diluted 1:5 were added to a 96-well ELISA plate pre-coated with an anti-human NLRP3 antibody. NLRP3 detection was carried out by incubation for 1h at 37 °C with a HRP-conjugate reagent, followed by the addition of a chromogen solution. OD values were read within 20 min using a Synergy HT microplate reader (BioTek^®^ Instruments, Winooski, VT, USA).

### 4.3. Micro Neutralization CPE-Based Assay

The Micro Neutralization (MN) assay performed in this study has been extensively described in previous publications [56,57]. Briefly, plasma samples were subjected to 2-fold serial dilutions in DMEM with 2% FBS (from 1:10 to 1:320, in duplicate) and added to two different 96-well plates. Following 1 h incubation at 37 °C with a viral solution containing 100 TCID50 of SARS-CoV-2, the virus-sample mixture was added to Vero E6 cells to assess whether the virus had retained its infectious capacity. After a 72 hour incubation, cells were inspected for signs of cytopathic effect to identify the highest sample dilution able to completely inhibit viral growth, i.e., the neutralization titer. Suitable negative and positive controls were also added to monitor the execution of the assay as well as the status of the plated cells and the virus itself.

### 4.4. miRNA Extraction and Analysis

Total RNAs were isolated from plasma samples using the miRNeasy Serum/Plasma Kit (Qiagen, Hilden, Germany) according to the manufacturer’s protocol. Briefly, 200 uL of thawed plasma were incubated with 1000 µL of QIAzol Lysis Reagent; then, samples were added with 200 µL of chloroform and 5 µL of ath-miR159a (6 pg of Spike-in). At the end of the procedure, miRNAs were eluted in 22 µL of nuclease-free water and stored at −80 °C until use. miRNA quality and integrity were assessed using the 2100 Bioanalyzer RNA system with the Pico Kit (Agilent Technologies, Santa Clara, CA, USA).

After miRNAs were converted to cDNA by reverse transcription using the TaqMan^®^ MicroRNA Reverse Transcription Kit (ThermoFisher Scientific, Waltham, MA, USA) and a specific custom RT pool, they were subjected to a 16-cycle pre-amplification step with a specific custom Preamp pool according to User Bulletin Publication Part Number 4,465,407 (Applied Biosystems, Waltham, MA, USA). The custom pool was made for the analysis of 14 target miRNAs (hsa-miR-101-3p, hsa-miR-106a-5p, hsa-miR-125a-5p, hsa-miR-126-3p, hsa-miR-132-3p, hsa-miR-137, hsa-miR-146a-5p, hsa-miR-155-5p, hsa-miR-195-5p, hsa-miR-210-3p, hsa-miR-21-5p, hsa-miR-221-3p, hsa-miR-223-5p, and hsa-miR-34a-5p), 2 endogenous controls (RNU48 and U6-RNA), and 1 exogenous control (ath-miR159a). These miRNAs were selected on the basis of the existing literature [52,58,59,60,61,62,63] and of previous studies conducted in our lab [64,65,66], showing their responsiveness to pro-inflammatory stimuli, and validated through the Non-coding RNAs in Inflammation (ncRI) database [67].

Then, samples were tested in triplicate on a customized OpenArray PCR miRNA Plate (Applied Biosystems, Waltham, MA, USA) to analyze miRNA expression levels by qPCR with the QuantStudio™ 12K Flex OpenArray^®^ Platform (Applied Biosystems Waltham, MA, USA). Briefly, the preamplification product of each sample was diluted 1/20 and mixed with 2X Open Array RT-PCR Master Mix (ThermoFisher Scientific, Waltham, MA, USA). The RT-PCR reaction mix was plated on an OpenArray PCR miRNA Plate using Accufill Systems (Applied Biosystems, Waltham, MA, USA) and run on QuantStudio™ 12K Flex PCR.

Data processing was carried out using the Expression Suite Software v.1.3 (Applied Biosystems), as detailed in [64]. qPCR quantifies miRNA expression in terms of relative threshold cycle (Crt) values and calculates (for each PCR reaction) an AmpScore as a qualitative parameter. Amplification curves with a no Crt value or one >27.5, or with an AmpScore <1.24 were set equal to the detection limit of 28. For statistical analysis, miRNA relative expression was calculated as Log_2_(RQ) [68], where RQ = Relative Quantification = 2^−ΔCrt^, and ΔCrt = Crt_miRNA_ − Crt_end_. Crt_miRNA_ is the mean Crt of the triplicate measure of each miRNA, while Crt_end_ is the mean Crt of the three endogenous controls. RNU48, U6-RNA, and ath-miR159a were chosen for normalization using the NormFinder algorithm [69], with a stability value of 0.10.

### 4.5. Statistical Analysis

Standard descriptive statistics were performed on all variables, reporting means with their standard deviation (SD) for continuous variables or frequencies with percentages for categorical variables. Obstetric adverse outcomes were analyzed both as a single event and combined into a composite adverse outcome. NLRP3 inflammasome, IgG, Microneutralization Assay titers, IgM, and IgA were evaluated at T0 and T1 as potentially associated with miRNA expression level. In addition, the miRNA expression level was quantified at T0 and T1.

We compared women with any positivity (i.e., IgG, IgM, or IgA) and negative ones by applying the t-test for continuous normally distributed clinical characteristics. Frequencies were compared with the Chi-Square or Fisher’s Exact test when the expected count was less than 5.

Associations between independent variables (inflammasome and microneutralization assay titers as continuous variables and IgA, IgM, IgG, and MN as categorical variables) and miRNA expression levels were evaluated by applying multivariable linear mixed models for repeated measures. The intercept was regarded as a random effect. Covariates were selected for potential inclusion in the multivariable models if they were associated with miRNAs in univariate analysis. Covariates associated with any miRNA in multivariable models were included in the final models, regardless of their significance, to control for confounding and obtain a unique model for all miRNAs. The final model was adjusted for time (first trimester or peripartum), gestational age at sample collection, and maternal age as fixed effects. The expression level of miRNA was obtained as log_2_(RQ). We estimated the β coefficient of the relationship between the continuous independent variable and miRNA and the percentage change (calculated as (2^(β)^ – 1) × 100) associated with one unit increase in the independent variable. For categorical independent variables (IgG, IgA, IgM, MN, or adverse outcomes), the coefficients and the percentage changes indicate the expression level of miRNA being in a category (positive or with an adverse outcome), versus the reference category (negative or absence of the adverse outcome). To take multiple testing into account, we performed multiple testing corrections, calculating the false discover rate (FDR) *p*-values using the Benjamini-Hochberg procedure.

First, we evaluated the association between NLRP3 inflammasome and miRNA expression levels, applying multivariable linear mixed models for repeated measures adjusted for time (T0, T1), gestational age at the sample, and maternal age. To investigate the modifying role of MN in the inflammasome-miRNA relationship, we added an interaction term to the previous model, and for miR-195-5p, which showed a significant interaction, we derived specific inflammasome-miRNA estimates for positive and negative MN. Marginal estimates were calculated at the mean level of continuous covariates (maternal age = 33.6 years, mean gestational age = 24.2 weeks) and selected reference levels of categorical variables (e.g., during peripartum).

We evaluated the estimate and percentage change of miRNA expression level of women who had an adverse outcome during pregnancy versus women who did not. We then investigated the modifying role of microneutralization in the adverse outcome-miRNA relationship, adding an interaction term to the previous model, and for miRNAs showing a significant interaction, we derived marginal means of miRNA expression level for positive and negative MN according to adverse outcome status, and we tested the difference between positive and negative MN in the group of women experiencing the adverse outcomes.

We also investigate the modifying role of the NLRP3 inflammasome in the adverse outcome-miRNA relationship by adding an interaction term (NLRP3×adverse outcome) and deriving specific inflammasome-miRNA estimates for women experiencing or not experiencing the adverse outcomes.

Statistical analyses were performed with SAS software (version 9.4).

### 4.6. Bioinformatic Analysis

All the bioinformatic analysis was performed using R software (v 4.0.4). MiRNA target analysis was conducted using the miRNAtap package by selecting genes that were present in at least 3 of the 5 datasets of miRNAtap. Then, target genes were compared with genes associated with inflammation/pregnancy outcomes (gestational diabetes, premature birth, and pre-eclampsia) as reported in DisGeNET datasets (v7.0) [70].

## 5. Conclusions

Pregnancy is often regarded as an immunological paradox, characterized by a delicate equilibrium between tolerance towards the fetus and protection against exogenous threats such as viral infections. In this study, we gained new insights into the complexity of such a balance and proposed pyroptosis as a novel mechanism possibly modulating the trajectory of pregnancy in women who have been infected by SARS-CoV-2 and healthy controls. Although further studies are warranted to shed light on the precise biological events underlying the link between COVID-19, pregnancy, and pyroptosis, we suggest that circulating miRNAs might be good candidate markers for immune dysregulation and the risk of APOs.

## Figures and Tables

**Table 1:** IgG, Microneutralization Assay titers, IgM, and IgA in the first trimester of pregnancy (T0) and peripartum (T1).

Test	First Trimester (T0) ^1^	Peripartum (T1)
	*n* (%)	*n* (%)
**Any Positivity (IgG, IgM, IgA)**	**40**	**97**
**Positive IgG antibodies**	40 (100%)	97 (100%)
High Positive	4 (10.0%)	9 (9.3%)
Positive	22 (55.0%)	51 (52.6%)
Low Positive	14 (35.0%)	37 (38.1%)
Microneutralization (n = 137)	27 (67.5%)	79 (81.4%)
Microneutralization assay titers		
10	13 (32.5%)	28 (35.4%)
20	8 (29.6%)	22 (27.8%)
40	5 (18.5%)	16 (20.3%)
80	1 (3.7%)	9 (11.4%)
160	0 (0.0%)	2 (2.5%)
320	0 (0%)	2 (2.5%)
**Positive IgM antibodies**	12 (30.0%)	52 (53.6%)
High Positive	0 (0%)	1 (1.0%)
Positive	3 (7.5%)	20 (20.6%)
Low Positive	9 (22.5%)	31 (32.0%)
**Positive IgA antibodies**	3 (7.5%)	23 (10.0%)
High Positive	0 (0%)	1 (4.3%)
Positive	0 (0%)	5 (21.7%)
Low Positive	3 (7.5%)	17 (23.7%)

^1^ Data at T0 for two subjects are missing.

**Table 2:** Association between NLRP3 inflammasome and miRNA expression levels. Estimates of miRNA expression level from a multivariable linear mixed model for repeated measures adjusted for time, gestational age at sampling, and maternal age as a fixed effect. The intercept was regarded as a random effect. Β_NLRP3_ is the change in miRNA expression level (calculated as log_2_(RQ)) associated with one unit increase in the NLRP3 inflammasome. Percentage change is calculated as (2^(β)^ − 1) × 100; * *p*-value of the interaction (NLRP3 × MN) for miR-195-5p, obtained from the previous model plus an interaction term between inflammasome and MN, and is *p* = 0.018. SE, standard error; LCI, lower confidence interval; UCI, upper confidence interval; MN, microneutralization; FDR, false discovery rate.

miRNA	β_NLRP3_ (SE)	Percentage Change	95% LCI	95% UCI	*p*-Value	FDR *p*-Value
miR-21-5p	−0.002 (0.031)	−0.1	−4.3	4.2	0.945	0.945
miR-34a-5p	0.135 (0.037)	9.8	4.4	15.5	**<0.001**	**0.002**
miR-101-3p	0.097 (0.029)	7	2.8	11.3	**0.001**	**0.002**
miR-106a-5p	−0.008 (0.055)	−0.6	−7.8	7.2	0.881	0.945
miR-125a-5p	0.132 (0.031)	9.6	5	14.4	**<0.001**	**<0.001**
miR-126-3p	0.093 (0.030)	6.7	2.4	11.2	**0.002**	**0.004**
miR-132-3p	0.046 (0.032)	3.2	−1.1	7.8	0.147	0.187
miR-146a-5p	0.095 (0.028)	6.8	2.8	11	**0.001**	**0.002**
miR-155-5p	0.103 (0.029)	7.4	3.2	11.7	**<0.001**	**0.002**
miR-195-5p	0.045 (0.048)	3.2	−3.4	10.2	0.346 *	0.404
miR-210-3p	0.052 (0.03)	3.6	−0.5	7.9	0.083	0.116
miR-221-3p	0.108 (0.031)	7.8	3.3	12.4	**0.001**	**0.002**
miR-223-5p	0.059 (0.028)	4.2	0.2	8.3	**0.039**	**0.061**

## Data Availability

All relevant data is contained within the article. The original contributions presented in the study are included in the article/supplementary material. Further inquiries can be directed to the corresponding author (VB).

## Supplementary Materials

The supporting information can be downloaded at: https://www.mdpi.com/article/10.3390/ijms24119278/s1.
