# Supplementary material for: Pyroptosis: A Promising Mechanism Linking SARS-CoV-2 Infection to Adverse Pregnancy Outcomes
Source: Int J Mol Sci. 2023 May 25;24(11):9278. doi: 10.3390/ijms24119278 (PMC10252646; doi:10.3390/ijms24119278)
Supplement: Supplementary file 1 [file ijms-24-09278-s001.zip › ijms-2324060-supplementary.pdf]

Supplementary materials

Pyroptosis: a promising mechanism linking SARS-CoV-2 infection to adverse pregnancy outcomes

Paola Monti [1], Giulia Solazzo [1], Veronica Accurti [2], Bianca Gambitta [2], Simona Iodice [1], Simona Boito [2], Laura Cantone [1], Alessandro Manenti [3], Laura Dioni [1], Emanuele Montomoli [3,4], Nicola Persico [2,5,6]†* and Valentina Bollati [1,6,7]†*

[1] EPIGET Lab, Department of Clinical Sciences and Community Health, University of Milan, Milan, Italy; paola.monti@unimi.it; giulia.solazzo@unimi.it; simona.iodice@unimi.it; laura.cantone@unimi.it; laura.dioni@unimi.it; valentina.bollati@unimi.it

[2] Fetal Medicine and Surgery Service, Fondazione IRCCS Ca' Granda, Ospedale Maggiore Policlinico, Milan, Italy; veronica.accurti@gmail.com; b.gambitta@gmail.com; simona.boito@gmail.com; nicola.persico@unimi.it

[3] VisMederi Srl, Siena, Italy; alessandro.manenti@vismederi.com; montomoli@vismederi.com

[4] Department of Molecular and Developmental Medicine, University of Siena, Siena, Italy; emanuele.montomoli@unisi.it

[5] Department of Clinical Sciences and Community Health, University of Milan, Milan, Italy; nicola.persico@unimi.it

[6] CRC, Center for Environmental Health, University of Milan, Milan, Italy; nicola.persico@unimi.it; valentina.bollati@unimi.it

[7] Occupational Health Unit, Fondazione IRCCS Ca' Granda Ospedale Maggiore Policlinico, Milan, Italy; valentina.bollati@unimi.it

† These authors contributed equally to this work and share last authorship.

* Correspondence: nicola.persico@unimi.it (NP); valentina.bollati@unimi.it (VB)

Citation: To be added by editorial staff during production.

Academic Editor: Firstname Lastname

Received: date
Revised: date
Accepted: date
Published: date

Copyright: © 2023 by the authors. Submitted for possible open access publication under the terms and conditions of the Creative Commons Attribution (CC BY) license (https://creativecommons.org/licenses/by/4.0/).

Supplementary Table S1. Clinical characteristics of 231 pregnant women, according to IgG, IgM or IgA positivity.

Characteristics	Mean ± SD or frequency (%)			p-value
	Negatives n=127	Any positivity n=104	All n=231	
Age, years	34.0 ± 4.6	33.2 ± 4.8	33.6 ± 4.7	0.218
Gestational age at sample, weeks	11.7 ± 0.6	11.7 ± 0.6	11.7 ± 1.0	0.619
**Anthropometric features**				
Maternal weight, Kg	61.4 ± 10.8	64.6 ± 13.4	62.8 ± 12.1	0.049
BMI, Kg/m²	22.5 ± 3.8	23.7 ± 4.3	23.1 ± 4.1	0.028
Categorical BMI				
Underweight (BMI < 18.5)	8 (6.3%)	4 (3.9%)	12 (5.2%)	
Lean (18.5 ≤ BMI < 25)	98 (77.2%)	69 (66.4%)	167 (72.3%)	0.048
Overweight (BMI ≥ 25)	21 (16.5%)	31 (29.8%)	52 (22.5%)	
**Smoking habits, n (%)**				
Never smoked	113 (89.0%)	94 (90.4%)	207 (89.6%)	
Stopped during pregnancy	7 (5.5%)	7 (6.7%)	14 (6.1%)	0.587
Smoker	7 (5.5%)	3 (2.9%)	10 (4.3%)	
**Features related to pregnancy**				
Parity				
Nulliparity	81 (63.8%)	67 (64.4%)	148 (64.1%)	0.919
Multiparity	46 (36.2%)	37 (35.6%)	83 (35.9%)	
Birth delivery mode, n(%)				
Spontaneous	49 (38.6%)	48 (46.2%)	97 (42.0%)	
Induction	52 (40.9%)	34 (32.7%)	86 (37.2%)	0.402
Cesarean	26 (20.5%)	21 (20.2%)	47 (20.3%)	
Previously spontaneous delivery				
16-30 weeks	2 (1.6%)	3 (2.9%)	5 (2.2%)	0.66
31-36 weeks	6 (4.7%)	4 (3.8%)	10 (4.3%)	1.0
37+ weeks	40 (31.5%)	34 (32.7%)	74 (32.0%)	0.846
Gestational age at delivery, weeks	39.4 ± 1.3	39.3 ± 1.3	39.3 ± 1.3	0.400
Conception method				
Spontaneous	123 (96.9%)	98 (94.2%)	221 (95.7%)	0.352
Fertilization technique	4 (3.1%)	6 (5.8%)	10 (4.3%)	
Type of fertilization technique (n=10)				
IVF	3 (75.0%)	2 (33.3%)	5 (50.0%)	
ICSI	0 (0.0%)	1 (16.7%)	1 (10.0%)	
Egg donation	1 (25.0%)	3 (50.0%)	4 (40%)	
**Anamnesis**				
Chronic Hypertension	*2 (1.6%)*	1 (1.0%)	3 (1.3%)	1.0
Systemic lupus erythematosus	0 (0.0%)	1 (1.0%)	1 (0.4%)	0.45
Antiphospholipid antibody syndrome	1 (0.8%)	1 (1.0%)	2 (0.9%)	1.0
Family history of Pre-eclampsia	4 (3.1%)	7 (6.7%)	11 (4.8%)	0.229
Mother affected	4 (3.1%)	6 (5.8%)	10 (4.3%)	
Sister affected	0 (0.0%)	1 (1.0%)	1 (0.4%)	
High risk of Pre-eclampsia risk bitest (≥1:150)	20 (15.7%)	16 (15.4%)	36 (15.6%)	0.919
Pre-eclampsia diagnosis	4 (3.15%)	2 (1.9%)	6 (2.6%)	0.693

Previous Small for Gestational Age (SGA) Newborn	2 (1.6%)	3 (2.9%)	5 (2.2%)	0.654
Delivery mode				
Caesarean section	45 (35.4%)	37 (35.6%)	82 (35.5%)	0.896
Vaginal	75 (59.1%)	61 (58.7%)	136 (58.9%)	
Ventouse	0 (0.0%)	1 (1.0%)	1 (0.4%)	
Vacuum	7 (5.5%)	5 (4.8%)	12 (5.2%)	
**Adverse outcome**				
Composite adverse outcome	14 (11.0%)	18 (17.3%)	32 (13.9%)	0.169
Pre-eclampsia	2 (1.6%)	3 (2.9%)	5 (2.2%)	0.660
Gestational diabetes	5 (3.9%)	6 (5.8%)	11 (4.8%)	0.549
Abnormal growth	8 (6.3%)	9 (8.7%)	17 (7.4%)	0.495
Small for gestational age	4 (3.2%)	2 (1.9%)	6 (2.6%)	0.693
Large for gestational age	4 (3.2%)	7 (6.7%)	11 (4.8%)	0.229
Delivery < 34 weeks	0 (0.0%)	1 (1.0%)	1 (0.4%)	0.450
**Neonatal parameters**				
Weight, gr	3321.0 ± 447.0	3301.7 ± 417.4	3312.0 ± 432.9	0.737
Sex				
Female	55 (43.3%)	47 (45.2%)	102 (44.2%)	0.774
Male	72 (56.7%)	57 (54.8%)	129 (55.8%)	
NICU admission	1 (0.8%)	3 (2.9%)	4 (1.7%)	0.329

Means of the two groups (Any positivity vs Negatives) were compared with t-test. Frequencies were compared with Chi-Square or Fisher's Exact test when the expected count was less than 5. IVF, In Vitro Fertilization; ICSI, Intra Cytoplasmic Sperm Injection; NICU, Neonatal Intensive Care Unit.

Supplementary Table S2. Mean NLRP3 concentrations measured at T0 and at T1, for women with and without APOs, for women with and without any positivity for Ig, and for women with and without positive MN assays.

Strata		Mean NLRP3	Standard Deviation	p-value
Time	T0	18.3	2.4	0.288
	T1	18.1	2.3	
Adverse outcome	No	18.3	2.4	0.615
	Yes	18.5	1.9	
Any positivity	Negative	18.3	2.3	0.938
	Positive	18.3	2.4	
MN	Negative	18.3	2.2	0.814
	Positive	18.2	3.3	

Means of the two groups (T0 vs T1; APOs No vs Yes; Any Ig positivity Yes vs No; MN Negative vs Positive) were compared with t-test.

Supplementary Table S3. Plasmatic miRNA levels between cases and controls.

miRNA	Means (95% CI) Any positivity n=104	Means (95% CI) Negatives n=127	p-value
hsa-miR-101-3p	0.12 (0.1 - 0.14)	0.13 (0.11 - 0.14)	0.510
hsa-miR-106a-5p	23 (17.1 - 31)	24.39 (18.65 - 31.9)	0.779
hsa-miR-125a-5p	0.17 (0.15 - 0.2)	0.19 (0.16 - 0.22)	0.528
hsa-miR-126-3p	20.04 (17.27 - 23.25)	24.34 (21.27 - 27.86)	0.058
hsa-miR-132-3p	0.4 (0.34 - 0.47)	0.41 (0.36 - 0.48)	0.724
hsa-miR-146a-5p	15 (13.05 - 17.23)	17.98 (15.85 - 20.4)	0.059
hsa-miR-155-5p	0.9 (0.78 - 1.05)	1.1 (0.97 - 1.26)	**0.047**
hsa-miR-195-5p	4.4 (3.3 - 5.7)	4 (3.1 - 5.2)	0.681
hsa-miR-210-3p	0.77 (0.66 - 0.89)	0.81 (0.71 - 0.93)	0.590
hsa-miR-21-5p	3.14 (2.68 - 3.69)	3.64 (3.15 - 4.21)	0.183
hsa-miR-221-3p	3.07 (2.64 - 3.57)	3.82 (3.33 - 4.38)	**0.037**
hsa-miR-223-3p	583 (507.2 - 670.1)	656.6 (578.6 - 745.1)	0.215
hsa-miR-34a-5p	0.04 (0.03 - 0.05)	0.04 (0.03 - 0.05)	0.805

Marginal means from ANCOVA models for repeated measures adjusted for time, maternal age and gestational week at blood examination. Dependent variable were log2(RQ), independent variable was the binomial classification of woman as negatives or any positivity (IgG, IgM, IgA) at the first trimester of pregnancy or peripartum.

Supplementary Table S4. Association between IgG positivity and miRNA expression level measured in the first trimester of pregnancy and peripartum

miRNA	First trimester			p-value	Peripartum			p-value
	β IgG+	95% LCI	95% UCI		β IgG+	95% LCI	95% UCI	
hsa-miR-21-5p	0.28	-0.2	0.76	0.249	0.16	-0.2	0.52	0.390
hsa-miR-34a-5p	0.03	-0.51	0.56	0.914	-0.10	-0.59	0.39	0.692
hsa-miR-101-3p	0.15	-0.29	0.6	0.501	-0.01	-0.38	0.36	0.956
hsa-miR-106a-5p	0.09	-0.71	0.9	0.818	0.16	-0.52	0.85	0.642
hsa-miR-125a-5p	0.1	-0.38	0.58	0.675	0.08	-0.32	0.48	0.702
hsa-miR-126-3p	0.26	-0.17	0.69	0.232	0.19	-0.22	0.6	0.356
hsa-miR-132-3p	0.05	-0.43	0.53	0.840	-0.08	-0.46	0.29	0.668
hsa-miR-146a-5p	0.34	-0.1	0.78	0.133	0.07	-0.28	0.42	0.678
hsa-miR-155-5p	0.15	-0.3	0.61	0.507	0.22	-0.14	0.57	0.230
hsa-miR-195-5p	-0.18	-0.93	0.58	0.647	-0.04	-0.64	0.55	0.890
hsa-miR-210-3p	0.03	-0.42	0.48	0.891	0.09	-0.27	0.44	0.639
hsa-miR-221-3p	0.29	-0.2	0.78	0.248	0.3	-0.09	0.69	0.128
hsa-miR-223-3p	0.33	-0.14	0.8	0.168	-0.03	-0.36	0.3	0.872

Estimates from a multivariable linear mixed model for repeated measures adjusted for time, gestational age at the sample, and maternal age. miRNAs are expressed as $\log_2$(RQ). β IgG+ is the estimated variation in miRNA expression level, in women with positive IgG, compared to negatives. The p-value refers to the difference in miRNA expression between positive and negative IgG. P-values controlled for multiple testing were not significant (FDR p-value>0.70) for all models.

Supplementary Table S5. Association between IgM positivity and miRNA expression level, in the first trimester of pregnancy and peripartum

miRNA	First trimester			p-value	Peripartum			p-value
	β IgM+	95% LCI	95% UCI		β IgM+	95% LCI	95% UCI	
hsa-miR-21-5p	0.12	-0.7	0.95	0.766	-0.04	-0.46	0.39	0.872
hsa-miR-34a-5p	0.10	-0.82	1.01	0.835	0.11	-0.47	0.68	0.719
hsa-miR-101-3p	0.21	-0.56	0.97	0.595	0.074	-0.357	0.504	0.736
hsa-miR-106a-5p	0.51	-0.86	1.89	0.463	0.05	-0.76	0.86	0.902
hsa-miR-125a-5p	-0.02	-0.84	0.8	0.958	0.3	-0.17	0.77	0.212
hsa-miR-126-3p	0.39	-0.34	1.13	0.296	0.41	-0.07	0.89	0.094
hsa-miR-132-3p	0.17	-0.64	0.99	0.676	0.21	-0.24	0.65	0.362
hsa-miR-146a-5p	0.31	-0.44	1.07	0.413	0.15	-0.26	0.56	0.474
hsa-miR-155-5p	-0.06	-0.84	0.72	0.88	0.26	-0.16	0.67	0.227
hsa-miR-195-5p	0.49	-0.81	1.79	0.458	-0.2	-0.9	0.5	0.57
hsa-miR-210-3p	0.63	-0.15	1.4	0.111	0.29	-0.13	0.71	0.178
hsa-miR-221-3p	0.01	-0.84	0.86	0.982	0.31	-0.14	0.77	0.179
hsa-miR-223-3p	0.75	-0.05	1.55	0.066	0.04	-0.35	0.42	0.857

Estimates from a multivariable linear mixed model for repeated measures adjusted for time, gestational age at the sample, and maternal age. miRNAs are expressed as $\log_2$(RQ). β IgM+ is the estimated variation in miRNA expression level, in women with positive IgM, compared to negatives The p-value refers to the difference in miRNA expression between positive and negative IgM. P-values controlled for multiple testing were not significant (FDR p-value>0.60) for all models.

Supplementary Table S6. Association between IgA positivity and miRNA expression level, in the first trimester of pregnancy and peripartum

miRNA	First trimester			p-value	Peripartum			p-value
	β IgA+	95% LCI	95% UCI		β IgA+	95% LCI	95% UCI	
hsa-miR-21-5p	-0.17	-1.78	1.43	0.831	0.02	-0.58	0.61	0.957
hsa-miR-34a-5p	0.17	-1.62	1.95	0.853	0.25	-0.55	1.06	0.535
hsa-miR-101-3p	-0.06	-1.54	1.43	0.938	0.12	-0.48	0.73	0.689
hsa-miR-106a-5p	-0.95	-3.63	1.73	0.486	0.16	-0.97	1.29	0.777
hsa-miR-125a-5p	0.32	-1.27	1.92	0.69	-0.08	-0.73	0.58	0.819
hsa-miR-126-3p	0.45	-0.98	1.89	0.537	0.34	-0.33	1.01	0.319
hsa-miR-132-3p	-0.16	-1.75	1.43	0.84	0.15	-0.47	0.78	0.624
hsa-miR-146a-5p	0.58	-0.89	2.05	0.44	-0.06	-0.64	0.52	0.841
hsa-miR-155-5p	0.10	-1.42	1.62	0.897	-0.21	-0.79	0.37	0.48
hsa-miR-195-5p	0.62	-1.91	3.14	0.632	0.25	-0.73	1.23	0.618
hsa-miR-210-3p	0.14	-1.37	1.65	0.858	0.39	-0.20	0.98	0.192
hsa-miR-221-3p	0.23	-1.42	1.88	0.785	-0.08	-0.72	0.57	0.811
hsa-miR-223-3p	0.45	-1.12	2.02	0.573	0.19	-0.36	0.73	0.502

Estimates from a multivariable linear mixed model for repeated measures adjusted for time, gestational age at the sample, and maternal age. miRNAs are expressed as log_2(RQ). β IgA+ is the estimated variation in miRNA expression level, in women with positive IgA, compared to negatives The p-value refers to the difference in miRNA expression between positive and negative IgA.

Supplementary Table S7. Association between MN positivity and miRNA expression level, in the first trimester of pregnancy and peripartum

miRNA-name	First trimester			p-value	Peripartum			p-value
	β MN+	95% LCI	95% UCI		β MN+	95% LCI	95% UCI	
hsa-miR-21-5p	0.39	-0.17	0.96	0.174	0.03	-0.35	0.41	0.874
hsa-miR-34a-5p	-0.23	-0.86	0.40	0.479	-0.02	-0.53	0.49	0.945
hsa-miR-101-3p	0.15	-0.38	0.67	0.585	-0.06	-0.44	0.32	0.773
hsa-miR-106a-5p	0.07	-0.88	1.02	0.887	-0.11	-0.82	0.60	0.764
hsa-miR-125a-5p	0.11	-0.46	0.67	0.712	0.08	-0.33	0.49	0.705
hsa-miR-126-3p	0.29	-0.22	0.79	0.262	0.13	-0.29	0.56	0.535
hsa-miR-132-3p	0.13	-0.43	0.69	0.641	-0.07	-0.46	0.32	0.724
hsa-miR-146a-5p	0.42	-0.10	0.94	0.111	0.08	-0.29	0.44	0.670
hsa-miR-155-5p	0.40	-0.14	0.94	0.142	0.13	-0.24	0.50	0.481
hsa-miR-195-5p	0.17	-0.73	1.06	0.711	0.14	-0.48	0.76	0.662
hsa-miR-210-3p	0.11	-0.43	0.64	0.696	0.04	-0.33	0.42	0.813
hsa-miR-221-3p	0.34	-0.24	0.93	0.244	0.26	-0.14	0.67	0.206
hsa-miR-223-3p	0.54	-0.02	1.09	0.057	0.05	-0.29	0.40	0.761

Estimates from a multivariable linear mixed model for repeated measures adjusted for time, gestational age at the sample, and maternal age. miRNAs are expressed as log_2(RQ). β MN+ is the estimated variation in miRNA expression level, in women with positive MN, compared to negatives The p-value refers to the difference in miRNA expression between positive and negative MN.

Supplementary table S8. Estimates and percentage change of miRNA expression level in women having an adverse outcome during pregnancy, versus women not experiencing it.

Adverse outcome (DV)	miRNA	β_{miRNA} (SE)	Percentage change	95% LCI	95% UCI	p-value	p-value adj
Composite adverse outcome	hsa-miR-21-5p	0.16 (0.23)	11.4	-18.5	52.2	0.498	0.871
	hsa-miR-34a-5p	0.18 (0.27)	13.7	-20.9	63.3	0.487	0.852
	hsa-miR-101-3p	0.11 (0.21)	8.2	-19.1	44.5	0.595	0.882
	hsa-miR-106a-5p	-0.01 (0.42)	-0.4	-43.9	76.9	0.990	0.990
	hsa-miR-125a-5p	0.01 (0.23)	0.4	-26.6	37.4	0.978	0.978
	hsa-miR-126-3p	0.17 (0.21)	12.6	-15.4	50.0	0.413	0.961
	hsa-miR-132-3p	0.10 (0.23)	6.9	-22.3	47.1	0.680	0.793
	hsa-miR-146a-5p	0.23 (0.20)	17.3	-10.7	54.2	0.251	0.877
	hsa-miR-155-5p	0.21 (0.21)	15.4	-13.5	53.9	0.330	0.769
	hsa-miR-195-5p	0.05 (0.39)	3.8	-39.3	77.6	0.891	0.891
	hsa-miR-210-3p	0.26 (0.21)	19.6	-10.5	59.8	0.224	0.784
	hsa-miR-221-3p	-0.01 (0.22)	-0.5	-26.2	34	0.972	0.972
	hsa-miR-223-3p	0.15 (0.20)	11.0	-15.4	45.7	0.449	0.786
Pre-eclampsia	hsa-miR-21-5p	-0.13 (0.54)	-8.5	-56.4	92.2	0.814	0.933
	hsa-miR-34a-5p	0.74 (0.63)	66.9	-29.0	292.6	0.239	0.836
	hsa-miR-101-3p	-0.34 (0.50)	-21	-60.2	56.9	0.499	0.882
	hsa-miR-106a-5p	-1.95 (0.99)	-74.1	-93.3	-0.3	**0.050**	0.116
	hsa-miR-125a-5p	-0.51 (0.54)	-29.7	-66.5	47.4	0.349	0.611
	hsa-miR-126-3p	-0.07 (0.50)	-4.7	-51.6	87.6	0.889	0.961
	hsa-miR-132-3p	-0.76 (0.55)	-41	-72.2	25.3	0.169	0.428

	hsa-miR-146a-5p	0.15 (0.48)	10.7	-42.2	111.7	0.759	0.885
	hsa-miR-155-5p	0.13 (0.50)	9.3	-44.9	116.7	0.798	0.882
	hsa-miR-195-5p	1.70 (0.93)	224.6	-8.3	1049.5	0.068	0.475
	hsa-miR-210-3p	0.20 (0.50)	14.8	-42.3	128.4	0.692	0.969
	hsa-miR-221-3p	-0.29 (0.52)	-18.3	-59.6	65.3	0.573	0.972
	hsa-miR-223-3p	-0.01 (0.47)	-0.6	-47.9	89.3	0.984	0.984
Gestational diabetes	hsa-miR-21-5p	0.70 (0.37)	62.1	-2.6	169.6	0.063	0.220
	hsa-miR-34a-5p	0.05 (0.44)	3.5	-43.1	88.2	0.910	0.910
	hsa-miR-101-3p	0.48 (0.35)	39.8	-13.1	124.9	0.166	0.882
	hsa-miR-106a-5p	1.52 (0.68)	187.3	13.2	628.8	**0.026**	0.093
	hsa-miR-125a-5p	0.49 (0.38)	40.0	-16.3	134	0.198	0.611
	hsa-miR-126-3p	0.55 (0.34)	46.4	-8.6	134.4	0.112	0.786
	hsa-miR-132-3p	0.51 (0.38)	42.3	-15.5	139.8	0.184	0.428
	hsa-miR-146a-5p	0.48 (0.33)	39.3	-11.1	118.3	0.147	0.877
	hsa-miR-155-5p	0.07 (0.35)	5.2	-34.6	69	0.834	0.882
	hsa-miR-195-5p	-0.83 (0.64)	-43.9	-76.6	34.5	0.194	0.680
	hsa-miR-210-3p	0.74 (0.35)	66.5	3.7	167.1	**0.035**	0.244
	hsa-miR-221-3p	0.06 (0.36)	4.0	-36.4	70	0.875	0.972
	hsa-miR-223-3p	0.49 (0.33)	40.3	-10.1	118.9	0.135	0.786
Abnormal growth	hsa-miR-21-5p	-0.32 (0.3)	-19.6	-46.8	21.3	0.296	0.692
	hsa-miR-34a-5p	0.17 (0.35)	12.2	-30.4	80.8	0.635	0.875
	hsa-miR-101-3p	-0.11 (0.28)	-7.1	-36.6	36.2	0.706	0.882
	hsa-miR-106a-5p	-1 (0.55)	-49.8	-76.3	6.3	0.072	0.125
	hsa-miR-125a-5p	-0.06 (0.3)	-4.2	-36.6	44.7	0.837	0.976

	hsa-miR-126-3p	0.01 (0.28)	0.9	-30.8	47.2	0.961	0.961
	hsa-miR-132-3p	-0.22 (0.31)	-14.1	-43.6	30.8	0.477	0.668
	hsa-miR-146a-5p	0.1 (0.26)	7.3	-25.2	54	0.700	0.885
	hsa-miR-155-5p	0.41 (0.28)	33.3	-8.8	94.7	0.137	0.478
	hsa-miR-195-5p	0.35 (0.52)	27.2	-37.4	158.2	0.504	0.738
	hsa-miR-210-3p	-0.13 (0.28)	-8.6	-37.6	34.1	0.645	0.969
	hsa-miR-221-3p	0.07 (0.29)	5.1	-29.0	55.7	0.802	0.972
	hsa-miR-223-3p	0.06 (0.26)	4.1	-27.3	49.1	0.825	0.962
SGA	hsa-miR-21-5p	-1.04 (0.49)	-51.3	-75.1	-4.7	**0.036**	0.220
	hsa-miR-34a-5p	1.15 (0.57)	121.7	2.0	381.8	0.044	0.311
	hsa-miR-101-3p	0.09 (0.46)	6.3	-43.2	99.1	0.847	0.882
	hsa-miR-106a-5p	-2.88 (0.89)	-86.4	-96.0	-54.4	**0.001**	0.009
	hsa-miR-125a-5p	0.57 (0.49)	48.6	-24.4	192	0.250	0.611
	hsa-miR-126-3p	0.26 (0.45)	19.6	-35.6	121.8	0.569	0.961
	hsa-miR-132-3p	-0.72 (0.5)	-39.5	-69.6	20.4	0.152	0.428
	hsa-miR-146a-5p	0.36 (0.43)	28.1	-29.1	131.4	0.411	0.885
	hsa-miR-155-5p	1.2 (0.45)	129.4	24.0	324.5	**0.008**	0.059
	hsa-miR-195-5p	0.73 (0.85)	66.2	-48.0	431	0.390	0.738
	hsa-miR-210-3p	-0.35 (0.46)	-21.7	-58.2	46.7	0.444	0.969
	hsa-miR-221-3p	0.66 (0.47)	58.4	-16.6	200.8	0.159	0.972
	hsa-miR-223-3p	0.41 (0.43)	32.7	-26.3	138.8	0.344	0.786
LGA	hsa-miR-21-5p	0.1 (0.37)	7.4	-35.2	78.1	0.781	0.933
	hsa-miR-34a-5p	-0.39 (0.43)	-23.9	-57.6	36.5	0.358	0.836
	hsa-miR-101-3p	-0.21 (0.34)	-13.5	-45.8	38.2	0.544	0.882

hsa-miR-106a-5p	0.11 (0.68)	7.9	-57.3	172.7	0.872	0.990
hsa-miR-125a-5p	-0.41 (0.37)	-25	-54.7	24.3	0.264	0.611
hsa-miR-126-3p	-0.12 (0.34)	-8.3	-42.2	45.6	0.713	0.961
hsa-miR-132-3p	0.07 (0.38)	5.1	-37.3	76.1	0.849	0.849
hsa-miR-146a-5p	-0.05 (0.32)	-3.2	-37.8	50.8	0.887	0.887
hsa-miR-155-5p	-0.05 (0.34)	-3.4	-39.5	54.0	0.882	0.882
hsa-miR-195-5p	0.12 (0.64)	8.8	-54.4	159.3	0.849	0.891
hsa-miR-210-3p	0.003 (0.34)	0.2	-37.3	60.2	0.994	0.994
hsa-miR-221-3p	-0.26 (0.35)	-16.6	-48.4	34.8	0.457	0.972
hsa-miR-223-3p	-0.14 (0.32)	-9.3	-41.5	40.8	0.663	0.928

Estimates (β_{miRNA}) and percentage change of miRNA expression level in the woman having an adverse outcome during pregnancy versus woman not experiencing it. Multivariable linear mixed models for repeated measures adjusted for time, gestational age at the sample, and maternal age. miRNAs are expressed as log_2(RQ). Percentage change is calculated as $(2^{(\beta)}-1)\bullet 100$.

Supplementary Table S9. Interaction of MN with adverse outcomes in the expression level of miRNAs (hsa-miR-125a-5p and hsa-miR-155-5p)

**miRNA**	**Adverse outcome**	**MN**	**Expression level of miRNA in women with/without adverse outcome log_2(RQ)**		**p-interaction** (MN* adverse outcomes)	**p-value raw (MN+ vs MN-)**	**p-value adj (MN+ vs MN-)**
			**Without**	**With**			
hsa-miR-125a-5p	Composite adverse outcome	MN-	-2.42 (-2.61, -2.24)	-2.69 (-3.16, -2.22)	**0.015**	**0.04**	0.161
		MN+	-2.66 (-2.98, -2.33)	-1.89 (-2.58, -1.19)			
	Pre-eclampsia	MN-	-2.44 (-2.61, -2.26)	-3.68 (-4.89, -2.47)	**0.016**	**0.02**	0.078
		MN+	-2.55 (-2.85, -2.25)	-1.22 (-3.03, 0.59)			
	Gestational diabetes	MN-	-2.47 (-2.64, -2.29)	-2.25 (-3.1, -1.39)	0.239	0.309	0.715
		MN+	-2.59 (-2.9, -2.28)	-1.6 (-2.67, -0.52)			
	Abnormal growth	MN-	-2.45 (-2.63, -2.27)	-2.55 (-3.19, -1.91)	0.794	0.885	0.999
		MN+	-2.52 (-2.83, -2.21)	-2.47 (-3.49, -1.45)			
	SGA	MN-	-2.47 (-2.65, -2.3)	-1.9 (-2.97, -0.82)	0.983	0.935	1
		MN+	-2.54 (-2.84, -2.23)	-1.98 (-3.79, -0.17)			
	LGA	MN-	-2.44 (-2.61, -2.26)	-2.91 (-3.69, -2.12)	0.732	0.805	0.995
		MN+	-2.51 (-2.81, -2.2)	-2.74 (-3.97, -1.51)			
hsa-miR-155-5p	Composite adverse outcome	MN-	0.04 (-0.13, 0.2)	0.01 (-0.43, 0.44)	**0.029**	0.09	0.309
		MN+	-0.2 (-0.5, 0.09)	0.62 (-0.01, 1.24)			
	Pre-eclampsia	MN-	0.04 (-0.12, 0.2)	-0.44 (-1.56, 0.68)	**0.033**	**0.044**	0.156
		MN+	-0.1 (-0.37, 0.18)	1.5 (-0.14, 3.14)			
	Gestational diabetes	MN-	0.04 (-0.12, 0.2)	-0.09 (-0.88, 0.7)	0.329	0.432	0.848
		MN+	-0.09 (-0.37, 0.19)	0.37 (-0.61, 1.35)			

	Abnormal growth	MN-	0.01 (-0.15, 0.17)	0.33 (-0.26, 0.92)	0.486	0.63	0.958
		MN+	-0.11 (-0.4, 0.17)	0.58 (-0.33, 1.48)			
	SGA	MN-	0.01 (-0.15, 0.16)	1.06 (0.08, 2.05)	0.544	0.621	0.953
		MN+	-0.1 (-0.38, 0.17)	1.52 (-0.1, 3.15)			
	LGA	MN-	0.04 (-0.12, 0.2)	-0.09 (-0.82, 0.64)	0.638	0.753	0.988
		MN+	-0.07 (-0.34, 0.21)	0.1 (-0.99, 1.2)			
hsa-miR-195-5p	Composite adverse outcome	MN-	2.03 (1.73, 2.34)	2.25 (1.48, 3.01)	0.268	0.318	0.732
		MN+	2.13 (1.68, 2.58)	1.77 (0.78, 2.75)			
	Pre-eclampsia	MN-	2.03 (1.75, 2.31)	3.45 (1.47, 5.43)	0.503	0.511	0.897
		MN+	2.01 (1.59, 2.42)	4.37 (1.72, 7.03)			
	Gestational diabetes	MN-	2.1 (1.81, 2.39)	1.15 (-0.2, 2.5)	0.676	0.676	0.973
		MN+	2.09 (1.67, 2.52)	1.47 (-0.1, 3.04)			
	Abnormal growth	MN-	2.01 (1.71, 2.3)	2.8 (1.76, 3.84)	**0.013**	**0.018**	0.075
		MN+	2.14 (1.71, 2.56)	1.15 (-0.26, 2.56)			
	SGA	MN-	2.03 (1.74, 2.31)	3.55 (1.76, 5.34)	**0.021**	**0.022**	0.087
		MN+	2.09 (1.68, 2.51)	0.4 (-2.24, 3.05)			
	LGA	MN-	2.04 (1.75, 2.33)	2.48 (1.19, 3.76)	0.134	0.147	0.435
		MN+	2.11 (1.68, 2.53)	1.31 (-0.38, 3)			

Estimated marginal means of miRNA expression level from a multivariable linear mixed model for repeated measures adjusted for time, gestational age at the sample, MN, adverse outcome, the interaction between adverse outcome and MN, and maternal age. miRNAs are expressed as log_2(RQ). The p-interaction refers to the significance of the interaction term (MN*adverse outcome). The p-value raw (MN+ vs MN-) refers to the difference between positive and negative microneutralization in the group of women experiencing the adverse outcomes. The p-value adj (MN+ vs MN-) refers to p-values adjusted for multiple comparisons.

Supplementary Table S10. Interaction of NLRP3 inflammasome with adverse outcomes in the expression level of miRNAs (hsa-miR-101a-3p, hsa-miR-126-3p, hsa-miR-132-3p, and hsa-miR-146a-5p)

**miRNA**	**Adverse outcome**		**expression level of miRNA in women with/without adverse outcome**	**p-value**	**p-value interaction (NLRP3* adverse outcome)**
hsa-miR-101-3p	Composite adverse outcome	No	0.11 (0.05, 0.17)	**<0.001**	0.129
		Yes	-0.05 (-0.25, 0.15)	0.604	
	Pre-eclampsia	No	0.1 (0.04, 0.16)	**0.001**	0.626
		Yes	-0.04 (-0.58, 0.51)	0.898	
	Gestational diabetes	No	0.1 (0.04, 0.16)	**0.001**	0.544
		Yes	-0.01 (-0.36, 0.34)	0.954	
	Abnormal growth	No	0.11 (0.05, 0.17)	**<0.001**	**0.046**
		Yes	-0.16 (-0.42, 0.1)	0.225	
	SGA	No	0.11 (0.05, 0.17)	**<0.001**	**0.033**
		Yes	-0.22 (-0.52, 0.08)	0.145	
	LGA	No	0.1 (0.04, 0.16)	**0.001**	0.678
		Yes	-0.02 (-0.6, 0.55)	0.934	
hsa-miR-126-3p	Composite adverse outcome	No	0.1 (0.04, 0.16)	**0.001**	0.781
		Yes	0.13 (-0.07, 0.34)	0.208	
	Pre-eclampsia	No	0.1 (0.04, 0.16)	**0.001**	0.063
		Yes	0.62 (0.07, 1.17)	**0.026**	
	Gestational diabetes	No	0.1 (0.04, 0.16)	**0.001**	0.706
		Yes	0.17 (-0.19, 0.53)	0.352	
	Abnormal growth	No	0.11 (0.05, 0.17)	**0.001**	0.795

		Yes	0.07 (-0.19, 0.33)	0.587	
	SGA	No	0.1 (0.04, 0.16)	**0.001**	0.969
		Yes	0.1 (-0.2, 0.39)	0.513	
	LGA	No	0.11 (0.05, 0.17)	**<0.001**	0.446
		Yes	-0.12 (-0.71, 0.46)	0.683	
hsa-miR-132-3p	Composite adverse outcome	No	0.06 (0, 0.13)	0.064	0.071
		Yes	-0.14 (-0.36, 0.07)	0.186	
	Pre-eclampsia	No	0.05 (-0.01, 0.12)	0.099	0.134
		Yes	-0.37 (-0.91, 0.18)	0.187	
	Gestational diabetes	No	0.04 (-0.02, 0.11)	0.193	0.816
		Yes	0.09 (-0.28, 0.45)	0.644	
	Abnormal growth	No	0.07 (0, 0.13)	**0.039**	**0.002**
		Yes	-0.38 (-0.66, -0.11)	**0.007**	
	SGA	No	0.06 (0, 0.13)	**0.049**	**0.005**
		Yes	-0.41 (-0.73, -0.09)	**0.012**	
	LGA	No	0.05 (-0.02, 0.11)	0.136	0.414
		Yes	-0.2 (-0.8, 0.39)	0.507	
hsa-miR-146a-5p	Composite adverse outcome	No	0.09 (0.03, 0.15)	**0.003**	0.471
		Yes	0.16 (-0.03, 0.36)	0.101	
	Pre-eclampsia	No	0.09 (0.04, 0.15)	**0.001**	0.149
		Yes	0.5 (-0.05, 1.04)	0.075	
	Gestational diabetes	No	0.09 (0.04, 0.15)	**0.001**	0.601
		Yes	0.18 (-0.15, 0.52)	0.285	
	Abnormal growth	No	0.1 (0.04, 0.15)	**0.001**	0.925
		Yes	0.11 (-0.15, 0.36)	0.403	

SGA	No	0.09 (0.04, 0.15)	**0.001**	0.854
	Yes	0.12 (-0.17, 0.41)	0.408	
LGA	No	0.1 (0.04, 0.15)	**0.001**	0.697
	Yes	-0.01 (-0.58, 0.55)	0.959	

Estimates of miRNAs expression levels from a multivariable linear mixed model for repeated measures adjusted for time, gestational age at the sample, NLRP3 inflammasome, adverse outcome, the interaction between adverse outcome and NLRP3 inflammasome, and maternal age as a fixed effect. The intercept was regarded as a random effect. miRNAs are expressed as log_2(RQ). The p-interaction refers to the significance of the interaction term (NLRP3 inflammasome*adverse outcome).

International Journal of
Molecular Sciences

MDPI

Supplementary Table S11. Number of target genes for each miRNA analyzed.

miRNA	N target genes
hsa-miR-101-3p	418
hsa-miR-125a-5p	305
hsa-miR-126-3p	10
hsa-miR-146a-5p	109
hsa-miR-155-5p	253
hsa-miR-195-5p	749
hsa-miR-221-3p	182
hsa-miR-223-5p	44
hsa-miR-34a-5p	464

Supplementary Table S12. Target genes associated with inflammation.

miRNA	EntrezID	Gene	UniProt	Protein Class
hsa-miR-101-3p	10019	*SH2B3*	Q9UQQ2	
	1906	*EDN1*	P05305	Signaling
	351	*APP*	P05067	Enzyme modulator
	3717	*JAK2*	O60674	Kinase
	5562	*PRKAA1*	Q13131	Kinase
	5743	*PTGS2*	P35354	Enzyme
	5806	*PTX3*	P26022	
hsa-miR-101-3p<br>hsa-miR-125a-5p<br>hsa-miR-34a-5p	6781	*STC1*	P52823	Signaling
hsa-miR-125a-5p	1236	*CCR7*	P32248	G-protein coupled receptor
	1978	*EIF4EBP1*	Q13541	Nucleic acid binding
	2056	*EPO*	P01588	
	3976	*LIF*	P15018	Signaling
	5770	*PTPN1*	P18031	
	6774	*STAT3*	P40763	Nucleic acid binding
hsa-miR-146a-5p	5354	*PLP1*	P60201	Cellular structure
	885	*CCK*	P06307	
hsa-miR-155-5p	10135	*NAMPT*	P43490	Signaling
	1557	*CYP2C19*	P33261	

	2252	*FGF7*	P21781	Signaling
	3091	*HIF1A*	Q16665	Transcription factor
	5970	*RELA*	Q04206	Transcription factor
	8651	*SOCS1*	O15524	Enzyme modulator
hsa-miR-195-5p<br>hsa-miR-223-5p<br>hsa-miR-34a-5p	2182	*ACSL4*	O60488	Enzyme
hsa-miR-195-5p	2247	*FGF2*	P09038	Signaling
	4088	*SMAD3*	P84022	Transcription factor
	54567	*DLL4*	Q9NR61	
	5979	*RET*	P07949	Kinase
	627	*BDNF*	P23560	Signaling
	7422	*VEGFA*	P15692	Signaling
	7533	*YWHAH*	Q04917	Chaperone
	8862	*APLN*	Q9ULZ1	
	9332	*CD163*	Q86VB7	Enzyme
	940	*CD28*	P10747	
	958	*CD40*	P25942	
hsa-miR-195-5p<br>hsa-miR-34a-5p	4092	*SMAD7*	O15105	Transcription factor
hsa-miR-221-3p	3084	*NRG1*	Q02297	Signaling
	4908	*NTF3*	P20783	Signaling
	7532	*YWHAG*	P61981	Chaperone

hsa-miR-34a-5p	1947	*EFNB1*	P98172	Signaling
	23411	*SIRT1*	Q96EB6	Epigenetic regulator
	2532	*ACKR1*	Q16570	G-protein coupled receptor
	2625	*GATA3*	P23771	
	343	*AQP8*	O94778	Ion channel
	40	*ASIC2*	Q16515	Ion channel
	5054	*SERPINE1*	P05121	Enzyme modulator
	50943	*FOXP3*	Q9BZS1	
	5228	*PGF*	P49763	Signaling

Supplementary Table S13. Genes target by the 3 miRNAs and associated with gestational diabetes, premature birth or pre-eclampsia.

**miRNA**	**Gene**	**Disease**
hsa-miR-125a-5p	*CYP24A1*	Gestational Diabetes
hsa-miR-125a-5p	*FLOT2*	Gestational Diabetes
hsa-miR-125a-5p	*DICER1*	Gestational Diabetes
hsa-miR-125a-5p	*ANPEP*	Gestational Diabetes
hsa-miR-125a-5p	*LNPEP*	Gestational Diabetes
hsa-miR-125a-5p<br>hsa-miR-155-5p<br>hsa-miR-195-5p	*RREB1*	Gestational Diabetes
hsa-miR-125a-5p	*SLC26A6*	Gestational Diabetes
hsa-miR-125a-5p	*SLC7A1*	Gestational Diabetes
hsa-miR-125a-5p	*STAT3*	Gestational Diabetes
hsa-miR-125a-5p	*VDR*	Gestational Diabetes
hsa-miR-125a-5p	*LIN28A*	Gestational Diabetes
hsa-miR-125a-5p	*MS4A3*	Gestational Diabetes
hsa-miR-155-5p	*NAMPT*	Gestational Diabetes
hsa-miR-155-5p	*GNAS*	Gestational Diabetes<br>Premature Birth<br>Pre-Eclampsia
hsa-miR-155-5p	*HIF1A*	Gestational Diabetes<br>Pre-Eclampsia
hsa-miR-155-5p	*SMAD2*	Gestational Diabetes

hsa-miR-155-5p	*RPS6KB1*	Gestational Diabetes
hsa-miR-155-5p	*TCF7L2*	Gestational Diabetes
hsa-miR-155-5p	*ZNF236*	Gestational Diabetes
hsa-miR-195-5p	*ADAMTS5*	Gestational Diabetes
hsa-miR-195-5p	*ADCY5*	Gestational Diabetes
hsa-miR-195-5p	*SLC30A8*	Gestational Diabetes
hsa-miR-195-5p	*FASN*	Gestational Diabetes
hsa-miR-195-5p	*FGF2*	Gestational Diabetes
hsa-miR-195-5p	*FGFR1*	Gestational Diabetes<br>Premature Birth
hsa-miR-195-5p	*COBLL1*	Gestational Diabetes
hsa-miR-195-5p	*GHR*	Gestational Diabetes
hsa-miR-195-5p	*GLS2*	Gestational Diabetes
hsa-miR-195-5p	*DLL1*	Gestational Diabetes<br>Premature Birth
hsa-miR-195-5p	*HTR2A*	Gestational Diabetes
hsa-miR-195-5p	*IGF1R*	Gestational Diabetes
hsa-miR-195-5p	*INSR*	Gestational Diabetes
hsa-miR-195-5p	*LIPE*	Gestational Diabetes
hsa-miR-195-5p	*MTHFR*	Gestational Diabetes<br>Premature Birth<br>Pre-Eclampsia
hsa-miR-195-5p	*PAPPA*	Gestational Diabetes

hsa-miR-195-5p	*MAPK8*	Gestational Diabetes
hsa-miR-195-5p	*PTCH1*	Gestational Diabetes<br>Premature Birth
hsa-miR-195-5p	*PTH*	Gestational Diabetes
hsa-miR-195-5p	*BDNF*	Gestational Diabetes
hsa-miR-195-5p	*SLC2A3*	Gestational Diabetes
hsa-miR-195-5p	*SLC6A4*	Gestational Diabetes
hsa-miR-195-5p	*SSR1*	Gestational Diabetes
hsa-miR-195-5p	*VEGFA*	Gestational Diabetes<br>Premature Birth<br>Pre-Eclampsia
hsa-miR-195-5p	*CASR*	Gestational Diabetes
hsa-miR-195-5p	*CDK5R1*	Gestational Diabetes
hsa-miR-195-5p	*APLN*	Gestational Diabetes<br>Pre-Eclampsia
hsa-miR-195-5p	*CD163*	Gestational Diabetes
hsa-miR-125a-5p	*SLC27A4*	Premature Birth
hsa-miR-125a-5p	*IL6R*	Premature Birth
hsa-miR-125a-5p	*PMM2*	Premature Birth
hsa-miR-155-5p	*KRAS*	Premature Birth
hsa-miR-155-5p	*TPRKB*	Premature Birth
hsa-miR-155-5p	*ACTA1*	Premature Birth
hsa-miR-195-5p	*CRKL*	Premature Birth

hsa-miR-195-5p	*COL24A1*	Premature Birth
hsa-miR-195-5p	*RBPJ*	Premature Birth
hsa-miR-195-5p	*KMT2A*	Premature Birth
hsa-miR-195-5p	*PEX12*	Premature Birth
hsa-miR-195-5p	*PEX13*	Premature Birth
hsa-miR-195-5p	*DLL4*	Premature Birth
hsa-miR-125a-5p	*IL16*	Pre-Eclampsia
hsa-miR-155-5p	*TDO2*	Pre-Eclampsia
hsa-miR-195-5p	*COMT*	Pre-Eclampsia
hsa-miR-195-5p	*SERBP1*	Pre-Eclampsia
hsa-miR-195-5p	*EED*	Pre-Eclampsia
hsa-miR-195-5p	*ACVR2A*	Pre-Eclampsia

Supplementary Table S14. Genes targeted by the 3 miRNAs and shared between the two groups (inflammation and pregnancy complications).

**miRNA**	**Gene**
hsa-miR-195-5p	*APLN*
hsa-miR-195-5p	*BDNF*
hsa-miR-195-5p	*CD163*
hsa-miR-195-5p	*DLL4*
hsa-miR-195-5p	*FGF2*
hsa-miR-155-5p	*HIF1A*
hsa-miR-155-5p	*NAMPT*
hsa-miR-125a-5p	*STAT3*
hsa-miR-195-5p	*VEGFA*
